# Scoping Review on the Multimodal Classification of Depression and Experimental Study on Existing Multimodal Models

**DOI:** 10.3390/diagnostics12112683

**Published:** 2022-11-03

**Authors:** Umut Arioz, Urška Smrke, Nejc Plohl, Izidor Mlakar

**Affiliations:** 1 Faculty of Electrical Engineering and Computer Science, The University of Maribor, 2000 Maribor, Slovenia; 2 Department of Psychology, Faculty of Arts, The University of Maribor, 2000 Maribor, Slovenia

**Keywords:** multimodal depression classification, scoping review, artificial intelligence, real-world data, mental health

## Abstract

Depression is a prevalent comorbidity in patients with severe physical disorders, such as cancer, stroke, and coronary diseases. Although it can significantly impact the course of the primary disease, the signs of depression are often underestimated and overlooked. The aim of this paper was to review algorithms for the automatic, uniform, and multimodal classification of signs of depression from human conversations and to evaluate their accuracy. For the scoping review, the PRISMA guidelines for scoping reviews were followed. In the scoping review, the search yielded 1095 papers, out of which 20 papers (8.26%) included more than two modalities, and 3 of those papers provided codes. Within the scope of this review, supported vector machine (SVM), random forest (RF), and long short-term memory network (LSTM; with gated and non-gated recurrent units) models, as well as different combinations of features, were identified as the most widely researched techniques. We tested the models using the DAIC-WOZ dataset (original training dataset) and using the SymptomMedia dataset to further assess their reliability and dependency on the nature of the training datasets. The best performance was obtained by the LSTM with gated recurrent units (F1-score of 0.64 for the DAIC-WOZ dataset). However, with a drop to an F1-score of 0.56 for the SymptomMedia dataset, the method also appears to be the most data-dependent.

## 1. Introduction

Depression is a global public health concern with an alarming rise in prevalence [1]. In fact, the global prevalence of depression was estimated at 28% in 2021 [2]. Furthermore, depression is one of the most common comorbidities in patients with severe physical disorders [3] (e.g., cancer, cardiovascular, neurological, and inflammatory disorders). Comorbid depression has a significant negative impact on a patient’s quality of life (QoL) [4,5,6] and is associated with poor adherence to medical treatments. Additionally, it involves higher rates of healthcare expenditure [3,7].

Although depression is common in comorbidity and multimorbidity patterns, it is often underestimated and greatly undertreated [8], even in higher-income countries [9]. Since depression is often associated with other clinical conditions such as anxiety and pain syndromes, the signs are often overlooked during daily medical care by professionals without specific training in mental health [10]. There are also structural barriers related to the availability, affordability, time constraints, and attitudinal factors of patients, e.g., non-treatment-seeking behavior [11], which significantly contribute to late diagnosis.

Smartphones and ‘mHealth’ apps can overcome several of the barriers mentioned above There is growing evidence that remote sign and symptom monitoring of various diseases and disorders can significantly improve health outcomes [12]. The ‘mHealth’ apps represent the foundation of digital biomarkers such as risk, diagnostic, monitoring, prognostic, predictive, or response markers [13]. Since measurements can be performed in real time, as a momentary assessment, these biomarkers can be less subjected to recall bias than retrospective questionnaires completed at the clinic [14]. The latter are also prone to reporting bias; for instance, self-reports of negative mood states experienced in the past tend to be exaggerated in a negative direction [15]. Moreover, social desirability and adherence can further distort the quality of the information provided by using self-report questionnaires [16]. Hence, psychiatry and other relevant fields may improve their screening processes and care by complementing subjective indicators (such as validated clinical questionnaires) with objective measures (such as those gathered via ‘mHealth’ apps) [17].

There is growing interest regarding the exploitation of observable cues [18], such as language use, speech, and facial expressions, in the screening and prediction of mental health problems [19]. It is a well-known fact that a variety of observable cues are affected by being in a depressed state [20,21]. Among non-verbal cues, facial muscle movements [22], slower pupil dilation [23], changes in vocal tract dynamics [24], and various other cues are characteristic of depression [22]. Since these cues are generated spontaneously, the use of AI-supported classification can contribute to improving the objectivity of the assessment and monitoring of the signs of depression [22] and other psychiatric disorders [25].

The main motivation of this scoping review was to identify and evaluate the existing algorithms for the classification of signs of depression from multiple observable cues, simultaneously expressed using the three conversational modalities [26]—language (i.e., verbal), speech (i.e., vocal), and facial expressions (i.e., visual)—to complement data gathered with self-report questionnaires. The work within this paper is driven to address the following research questions: (Q1) “What artificial models for depression classification analyse all three groups of observable cues?” (Q2) “How do the multimodal algorithms perform compared to unimodal and bimodal implementations?” (Q3) “How, if at all, does the use of multiple multimodalities mitigate the performance degradation in unfavorable datasets?” While the findings of our review represent an important contribution to the literature in the broadest sense, they will also be applied in digital interventions implemented in the multicentric single-case experimental prospective study of the PERSIST project [27]. The overall objective of PERSIST is to leverage the potential of big data and advanced data analytics, which can improve cancer survivors’ treatment and follow-up decision-making and engagement. PERSIST is particularly concerned with patient-reported outcomes, patient satisfaction scoring, identifying high-risk patients and giving them the appropriate treatment, and supporting patients who are at high risk of reoccurrence. Depression classification algorithms, the central topic of this review, will be used in PERSIST to derive complementary information about the mental health of patients and to offer personalized feedback by analyzing the videos of diary recordings, which will be recorded by patients via smartphone. We build our research on the assumption that depression is likely expressed through all three modalities of communication continuously and simultaneously [18]. Hence, the prevailing unimodal and bimodal approaches to the AI-based recognition of signs of depression may be inadequate [28] Depression has a complex structure in general due to having a wide array of potential symptoms that can manifest slightly differently for each individual, depending on their current and past personal psychosocial context. Thus, the fusion of multimodal features is needed to create a discriminative and explanatory approach able to deal with the complex nature of depression. However, to our knowledge, the existing reviews have focused on biochemical studies [29], mental health applications and monitoring systems [8,30,31], and single observable cues or bimodal approaches [22,25,32]. Furthermore, the existing studies have revealed relatively high accuracy [31,33]. However, they should only be considered as proof-of-concept studies, without addressing any specific issues such as decision errors, data bias and transparency, and other ethical concerns. The data have the main role as far as biases in the process are concerned. Decision errors (especially false negatives) and data bias are some of the main issues of machine learning algorithms. From a clinical perspective, those biases could lead to improper clinical decisions and may cause severe consequences. The main sources of unwanted biases are either choosing the wrong subjects for the training dataset or the wrong factors for training. In any case, both situations may cause fatal errors and challenges in medical situations [34].

The outline of this scoping review is as follows. The six-stage methodological framework is introduced in Section 2. The illustration and analyses of the experimental results are presented in Section 3. Finally, the key findings, challenges, and limitations are explained in Section 4.

## 2. Materials and Methods

### 2.1. Overview

In the preparation of the study, we followed the six-stage methodological framework for scoping reviews outlined by Arksey and O’Malley [35], as follows: identifying the research questions; searching for relevant studies; selecting the relevant studies; charting the data; collating, summarizing, and reporting the results; consulting the study findings. PRISMA-ScR (Preferred Reporting Items for Systematic Reviews and Meta-Analyses—Extension for Scoping Reviews) [36] guidelines were followed to guarantee that the scoping review was systematic, transparent, and complete.

Beyond the scoping review framework, we applied the identified algorithms (with the available code) to two depression datasets to evaluate the accuracy of the AI-based processing of multimodal features for the classification of depression. The performance of the algorithms was evaluated using standard metrics.

### 2.2. Research Questions

Our scoping review was guided by these research questions derived from the main aim of the review:

- To what extent do the papers include the different types of modalities for depression classification?
- What types of algorithms currently exist for depression classification for observable cues and what is the performance of those algorithms?
- What is the effect of data bias on the results of existing algorithms and the contributions of the multimodal approach to decrease the bias?

### 2.3. Search Strategy

Four large and commonly used databases, Web of Science (WOS), IEEE Xplore, SCOPUS, and ACM Digital Library, were used to identify the relevant papers. After a preliminary search in all databases, which helped us to refine the search strategy and ensure that the databases provided adequate coverage of the research topic, we conducted the main search between 14 October and 21 October 2021.

Our search strategy combined terms related to depression (depression, depressed), cues (cue, feature, indicator, marker, sign, signal, symbol, pattern, style, clue, manifestation, expression), features (“feature extraction”, speech, text, audio, video, face, visual), emotion (emotion, “emotion recognition”), algorithms (“explainable AI”, “deep learning”, “machine learning”, supervised, unsupervised), and data fusion (“feature fusion”, “data fusion”, “decision fusion”) (Box 1).

Box 1 **The exact search string for Scopus.**

TITLE-ABS-KEY (“Depression”) AND TITLE-ABS-KEY (“Feature extraction” OR speech OR text OR audio OR video OR face OR visual) AND TITLE-ABS-KEY (cue OR feature OR indicator OR marker OR sign OR signal OR symbol OR pattern OR style OR clue OR manifestation OR expression OR emotion OR “emotion recognition” OR “Feature fusion” OR “data fusion” OR “decision fusion”) AND TITLE-ABS-KEY (“Explainable AI” OR “Deep learning” OR “machine learning” OR “supervised” OR “unsupervised”) AND (LIMIT-TO (LANGUAGE, “English”)) AND (LIMIT-TO (DOCTYPE, “ar”) OR LIMIT-TO (DOCTYPE, “cp”) OR LIMIT-TO (DOCTYPE, “ch”))

The inclusion criteria (being available in English and being published, i.e., pre-prints and other unpublished papers were not considered) were derived from the research questions guiding this scoping review and were set a priori. The exclusion criteria included non-technical papers including reviews, studies that included human participants who suffered from other disorders that affect behavior and displays of emotions (i.e., dementia, Parkinson’s disease, autism, schizophrenia, Alzheimer’s disease, neurological disorder, stroke, Huntington’s disease, paralysis, autism, multiple sclerosis, cerebral palsy, Down syndrome), studies that focused on psychological disorders other than depression (e.g., anxiety, insomnia), and studies that offered classifications based on other observable features of depression (i.e., ECG, EEG, fMRI). Additionally, for the qualitative synthesis or experimental part of the paper, studies that included algorithms not operating over all three channels of communication simultaneously were excluded.

### 2.4. Study Selection

**Figure 1:**
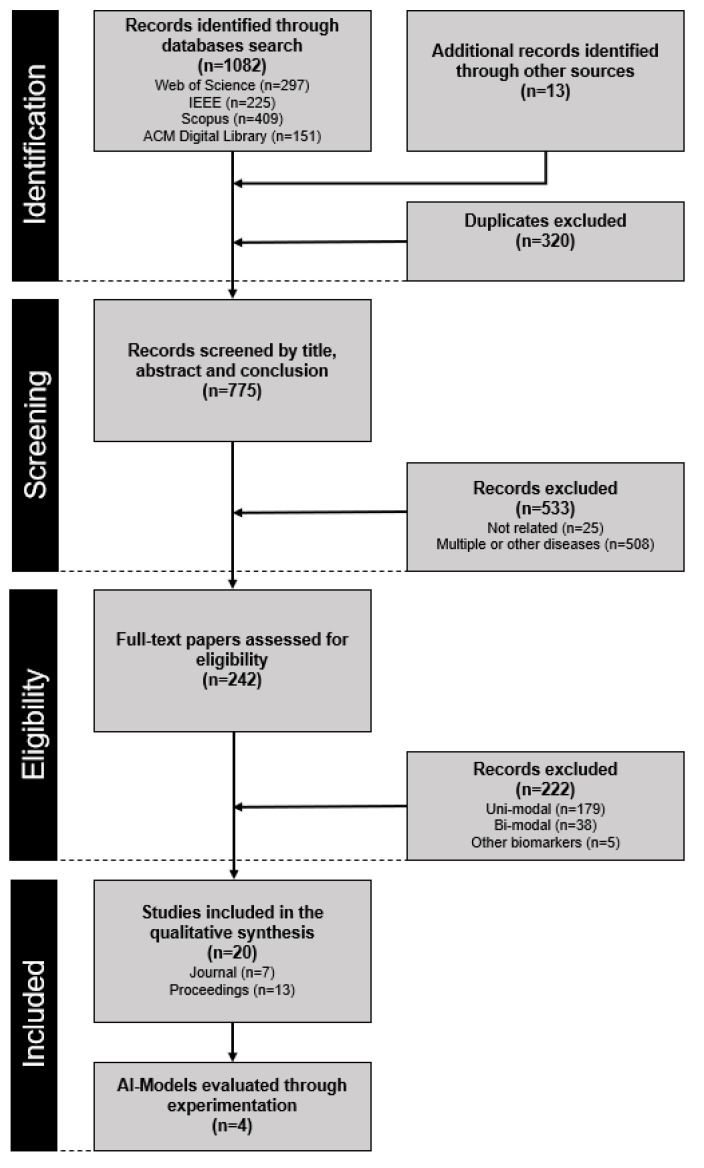
Flowchart of the search and study selection process.

All citations identified in the electronic databases were exported to Excel spreadsheets. The database searches yielded a total of 1082 papers, and 13 additional papers were identified through other sources (Figure 1). After duplicates were removed, 775 titles and abstracts were screened in three stages. In the first stage, the authors (I.M. and U.A.) individually excluded highly irrelevant papers. In the second stage, all authors (U.A., I.M., U.S., and N.P.) independently reviewed the titles and abstracts of the remaining citations and settled disagreements through discussion. After this step, 245 papers underwent a thorough full-text review independently by two authors (I.M. and U.A.). Disagreements in this phase were settled through discussion and adjudication by the remaining authors (U.S. and N.P.). In the end, 20 papers fulfilled the pre-determined criteria and were included in the scoping review. In the 20 papers, we identified four different approaches that processed the three communication channels simultaneously and corresponding pre-trained models that were found on public repositories (i.e., GitHub). The four approaches were implemented and evaluated by three authors (I.M., U.A., and U.S.) in experiments using DAIC-WOZ [37] and Media datasets [38] to assess the data bias.

### 2.5. Data Collection and Charting

We developed a spreadsheet to determine variables to be extracted from the reviewed papers. Two authors (I.M. and U.A.) then extracted the following data from each paper, as follows: (1) authors; (2) year of publication; (3) type of paper; (4) targeted diseases; (5) exclusion criteria; (6) modality (e.g., text, audio, visual cues); (7) AI/ML technique (e.g., CNN, LSTM, RF, SVM). During this process, the results were categorized into the following categories: (1) unimodal; (2) bimodal; (3) multimodal. The processes for data extraction and the categorization of results were refined and updated in an iterative process as the papers were reviewed. The resulting chart was used for the analysis.

### 2.6. Collating, Summarizing, and Reporting Results

We did not follow a well-structured meta-analytic approach in comparing and summarizing the results given the aim and nature of the scoping review. Since the aim of a scoping review includes mapping the existing findings on a topic, providing a descriptive overview, and (re)evaluating the algorithms on a proprietary dataset [39], the results were analyzed by I.M. and U.A. using a thematic analysis [40] for the multimodal category. To maintain rigor in collating and summarizing the results, this process was reviewed by U.S. and N.P.

### 2.7. Consultation Exercises

The review protocol of the study was developed with the help of the expertise from psychological (U.S., N.P.), technological (I.M., U.A.), and methodological (I.M., U.S., N.P.) fields. Relevant inclusion and exclusion criteria (e.g., specific conditions or comorbidities that could impact the expression of depression) were determined by consulting the healthcare professionals in the PERSIST and HosmartAI project.

## 3. Results

### 3.1. Study Characteristics of Scoping Review

**Figure 2:**
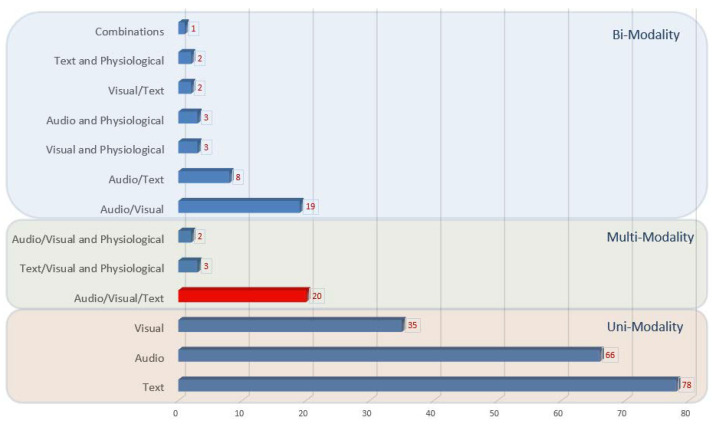
The number of papers per observable cue after the initial screening process.

Out of 242 eligible papers indicated after screening phase, 179 papers described unimodal algorithms (Figure 1), processing only text (*n* = 78), only acoustic cues (*n* = 66), or only visual cues (*n* = 35). 38 papers were found processing two communication channels simultaneously (i.e., bimodal algorithms); most of them considered acoustic and visual cues (*n* = 19), followed by text and acoustic cues (*n* = 8), acoustic and physiological cues (*n* = 3), visual and physiological cues (*n* = 3), text and visual cues (*n* = 2), text and physiological cues (*n* = 2), and different combinations of two modalities (*n* = 1). Twenty-five papers were found to process all three modalities, out of which 5 papers processed audio and visual cues alongside physiological biomarkers and 20 papers simultaneously processed language, speech, and visual cues (Figure 2). Since we were interested in algorithms capable of analyzing symptoms of depression from videos of diary recordings, we excluded those processing physiological biomarkers and those that did not process all of the modalities. The final 20 selected papers are summarized in Table 1.

#### 3.1.1. Data and Input Pre-Processing

Different modalities require different types of or approaches to input pre-processing. The most widely used techniques for data pre-processing are the linear discriminant analysis (LDA), synthetic minority oversampling technique (SMOTE), principal component analysis (PCA), and hidden Markov models (HMM) [60]. The LDA is used to remove redundant features by transforming them from a spatial space onto a lower-dimensional space. Using the LDA, the dataset can retain the most important features and can achieve higher class separability [61]. The PCA is an alternative technique to reduce the dimensionality of the datasets [62,63]. The PCA rotates the features in a way that possibly correlated features are transformed into a set of linearly uncorrelated features; that is, when orthogonal rotation is applied [64]. The SMOTE is used to generate a balanced class distribution by using an underrepresented dataset with synthetic patterns [65]. Most existing datasets, including DAIC-WOZ, are unbalanced, with depressive samples significantly being underrepresented [66]. HMMs are probabilistic models that are used to explain or derive the probabilistic characteristics of any random process. Using HMMs, the input data can be modelled as a series of outputs generated by multiple internal states [67]. As an alternative, evolutionary algorithms [64] and unsupervised autoencoders [68] were deployed to further optimize the datasets.

#### 3.1.2. Feature Extraction

Feature selection represents a step in the process in which a subset of variables (features) is selected to most accurately predict the targeted variable. Among the more popular approaches are the clustering (e.g., K-means or its variations), particle swarm optimization (PSO), RELIEFF, multivariate processing, and Boruta algorithm approaches [69]. The K-best approach is a K-nearest neighbor-based clustering feature selection technique. It is non-parametric and univariate in nature [69]. It is used to select K-best features from the feature set using an univariate statistical test. Particle swarm optimization is a computational technique that is used for optimizing non-linear functions, clustering, and feature selection [70]. PSO-based clustering and wrapper-based PSO were, for instance, proposed to improve the discrimination ability of the extracted speech features and to enhance the accuracy of the speaker-independent multiclass emotion recognition process by selecting only discriminative features [71]. RELIEFF [72] is one of the most successful filtering feature selection methods. In the algorithm, the quality of a feature is based on its capacity to differentiate samples that are near to each other. Minimum redundancy and maximum relevance (mRMR) is a multivariate processing approach where features are ranked based on their relevance to the target variable. The feature with the maximum relevance and minimum redundancy gets the highest rank in the mRMR process [73]. The Boruta algorithm is build based on a random forest classifier [74]. It works based on an iterative methodology to remove irrelevant attributes.

#### 3.1.3. AI/ML Techniques in the Reviewed Studies

CNN: In this review, it was found that the CNN technique is the most preferred AI/ML technique for the classification of depression (in 7 studies). CNN is one of the feed-forward NNs that can be used for many different fields (clustering, classification, regression, prediction, optimization, etc.). The CNN architecture is successfully applied in different ways to many speech recognition, emotion, and depression classification applications because of having powerful properties for capturing mixed or multimodal non-linear relationships among variables. Within our reviewed papers, [41,43] used a CNN as a depression estimation module for hand-crafted features, [46] used deep neural networks with attention fusion networks for the estimation of PHQ-8 scores, [50] proposed a hybrid deep learning model for different types of inputs and fused the outputs in a fusion network, and [58] used a causal CNN structure to model long sequences of patient video recordings.

SVM: The SVM technique is one of the most popular methods for depression classification due to having well-fitted binary issues of high dimensionality and suitability for non-linear kernel function settings [75]. Within our reviewed papers, [41] used an SVM as a classifier for psychoanalytic symptom presence identification, [28,45,53] built a depression detection model on an SVM as one of the regression models, and [57] investigated the different fusion types with SVMs.

RF: The RF technique obtains a result by using the collection of outputs of multiple decision trees. The main advantages of the RF technique are its easy usage and adaptation flexibility for both classification and regression issues. Within our reviewed papers, [28,41] used the RF technique for depression classification modeling, [42] translated features into predictive scores via RF, and [56] predicted the PHQ-8 scores an using RF model.

LSTM: LSTMs are used mainly for retaining information for longer periods for both classification and regression, but especially for sequence prediction issues. In a bidirectional LSTM (BiLSTM), each training sequence is applied in both directions (forward and backward) to separate recurrent nets. If you are using LSTM with multimodal features, the contribution of text features can be diminished via audio and visual modalities. To overcome this problem, a gating mechanism is used to control the contribution of each modality to the last decision (for more details, see Section 3.2.3). Within our reviewed papers, [43] utilized an LSTM by processing long-term dependencies, recognizing context information, and dealing with recent information; [44,51,52] used a BiLSTM for depression recognition; [59] utilized an LSTM to predict PHQ-8 scores; and [52] applied a gating mechanism to their network.

DT: The decision tree is one of the most popular and powerful algorithms for both classification and regression. Its structure is similar to a tree’s structure in that the branches show the results of the test and the leaves show the labels. Within our reviewed papers, [49] used predicted PHQ-8 scores and participant characteristics to construct decision tree, while [55] used a decision tree for multimodal fused features to classify the presence of depression.

MV-R: Multivariate regression is mainly used when the model has more than one response and one explanatory variable. Multivariate regression is used as a supervised machine learning algorithm and can be applied to different scientific disciplines. Within our reviewed papers, [48] used the Gaussian staircase model to generalize the distributions to be used in the binary classification of depression via multivariate regression.

#### 3.1.4. Early or Late Fusion

Fusion involves joining information from at least two or more different modalities such as audio, text, and video into a consistent representation to predict the assessment result where there are complementary expressions such as depression. Fusing different modalities increases the chance of obtaining higher precision by overcoming the shortages of individual modalities or interactions of multimodal features. All automatic depression classification models have five common stages, namely the input, pre-processing, feature extraction, machine learning, and output stages. Fusion can be applied either at the feature level (early fusion) or at the decision level (late fusion). In early fusion (feature level), multimodal features are involved in a merged feature set to be fed into the machine learning algorithms. Early fusion has different opportunities to be applied before or after the dimensionality reduction step in the feature extraction stage [76,77,78]. In late fusion (decision level), the results of different machine learning algorithms are combined and processed with a decision fusion algorithm to obtain the final decision.

In our scoping review, 9 papers (45%) applied late fusion, 6 papers (30%) applied early fusion, and 5 papers (25%) applied hybrid fusion (early+late) in their assessments. Early fusion was applied mainly via feature selection via an importance factor, continuing with only the most informative ones and concatenating them into one vector (fusion) for the classification process. As a different strategy, [51] used an attention fusion network and [56] used the bidirectional LSTM method for the fusion of the features. Late fusion was implemented with different approaches such as the winner-takes-all strategy (taking the decision of the single modality with the highest confidence score) [42], ensemble prediction (where depression is classified if one of the modalities predicts the subject as being depressed) [43], and the temporal framework using a simple feed-forward NN [44] and two hierarchical BiLSTM designs for the integration of all features [51].

### 3.2. Experimental Study

Following the scoping review, we carried out complementary experiments on two different depression datasets: DAIC-WOZ [37] and SymptomMedia [38]. In this section, we describe the datasets, the list of the multimodal features, and the feature extraction process. Then, we explain the implementation of AI models with performance metrics (F1-score, precision, and recall) and error metrics (MSE, RMSE, and MAE). Finally, we conclude the section with the results of the depression classification process.

#### 3.2.1. Datasets

DAIC-WOZ Database: This database is part of a larger clinical interviews corpus, the Distress Analysis Interview Corpus (DAIC), which is used for different types of psychological disorders, such as anxiety, depression, and post-traumatic stress disorder. A virtual agent (called Ellie and controlled by a person in a different place) was used in communicating with the patients to identify both verbal and non-verbal signs of psychological disorders [79]. There are 189 sessions and the training, development, and testing sets are pre-defined. Those sets include information about patient IDs, PHQ-8 scores, binary labels for PHQ-8 >= 10, and gender, and each session involves the transcript of the interaction, an audio file, audio features, and facial features.

SymptomMedia Dataset: This dataset contains guided film case studies about mental health (of diagnoses within the DSM-5 and ICD 10 classification systems), which is mainly used for continuing education for medical students, doctors, psychologists, counsellors, and other health care professionals. It contains more than 600 films of actors (actual patients were not used in the production of these films). The durations of the film range from 4 to 14 min. The recordings include only the appearance of the patient, while the interviewer is hidden. Moreover, a transcript is available for each film (for both interviewer and patient).

For our study, we selected 14 SymptomMedia recordings of individuals (i.e., professional actors) simulating different diagnoses of depression and 14 recordings of individuals simulating other mental health-related diagnoses that have a low probability of co-occurring together with major depression compared to other disorders [80,81] (Table 2). In total, 28 videos were selected to ensure an evenly balanced test set.

#### 3.2.2. Multimodal Feature Extraction

To evaluate the AI models, the following features were extracted from the SymptomMedia films (Table 3).

**Figure 3:**
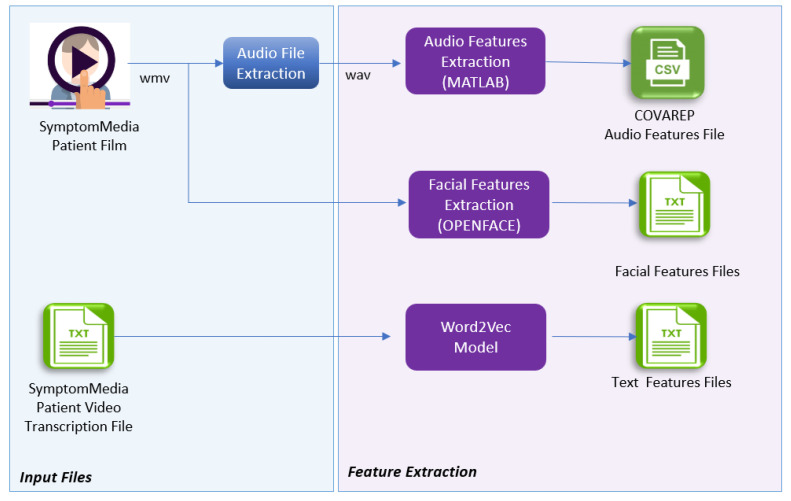
Feature extraction pipeline.

The feature extraction was applied only for the SymptomMedia dataset because the DAIC-WOZ dataset provides the extracted features within the dataset. For the SymptomMedia dataset, the audio file was extracted from the video via the Ffmpeg multimedia framework [82] as a first step. Then, the audio features were extracted with the COVAREP (Collaborative Voice Analysis Repository) tool [83] using MATLAB [84] software; the visual features were extracted using the OpenFace toolkit [85] and the Word2Vec model [86] was used to transform the words in the transcription file into a 300-feature vector representation (sentences x words x 300 features) for text feature extraction (Figure 3).

#### 3.2.3. AI Models and Evaluation Metrics

Based on Box 1, we selected SVM + RF with SVM late fusion, SVM + RF with RF late fusion, LSTM without gating, and LSTM with gating as models to be further investigated. To evaluate the models, we adapted the implementation proposed in [52] and related the code available via the GitHub repository [87]. In the following sections, we outline the implementation of each of the models.

SVM and RF: The SVM tries to predict the final output as an optimization problem. In that problem, it explores the best hyperplanes that maximize the size of the margin between two groups. By using the kernel function (*K*), an algorithm can easily find the most suitable hyperplane in a transformed feature space. In our case, the decision function for the test (*x*) has the following equation:

(1) $$g\left(x\right)=\underset{i}{\sum }\alpha_{i}y_{i}K\left(x_{i},x\right)-b\;\;\;\;\;\;\;\;\;\;\;\;\;\;\;\;$$

where *g*(*x*) is the decision function, α is the learned weight constant, y is the class label, K is the kernel function used for training (*x_i_*) and testing (*x*) the sample, and b is the learned threshold value.

On the other hand, the random forest (RF) ensembles the decision trees using the bagging method (a combination of learning models). In this way, the RF gives more accurate and stable predictions. There are mainly four steps for an RF:

1. Take *n* number of random records from the dataset;
2. Construct decision trees for each sample;
3. Generate an output for each tree;
4. Apply majority voting or averaging for the final output.

**Figure 4:**
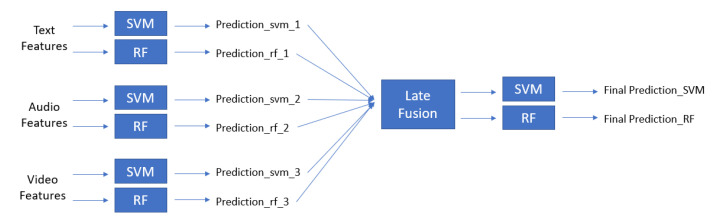
SVM and RF model architecture.

Here, we wanted to show the different effects of SVM and RF on late fusion. Each modality was classified separately using SVM and RF models and then the late fusions were performed by applying another SVM or RF model to predict the values (Figure 4).

LSTMs with or without gating: The gating mechanism is used for learning the effects of visual and auditory modalities on lexical information. This mechanism is important for determining the noise quantity while combining the three modalities with different effects to obtain the final result. The main motivation for using the gating mechanism was to overcome the undermining problem with the learned representation of text using other modalities. By using a gating mechanism, the contributions of visual and auditory modalities to the final result can be controlled [52].

The model includes feed-forward highway layers [88] for use with the gating mechanism for audio and video features. Those layers have two non-linear transforms (the ‘carry gate (Cg)’ to measure the transformation degree and ‘transform gate (Tg)’ to determine the amount of information to move forward).

The definitions of the gates are as follows:

(2) $$Transform\;Gate:\;T_{g\;}=\sigma \left(W_{T_{g}}I_{t}+b_{T_{g}}\right)\;\;\;$$

where *W* is the weight matrix and b is the bias vector:

(3) $$Carry\;Gate:\;C_{g}=1-T_{g}$$

The input vectors (It) are controlled by those two gates at each layer (feed-forward layer, *H*), as follows [52]:

(4) $$y=T_{g}\;.\;H+C_{g}\;.\;I_{t}$$

**Figure 5:**
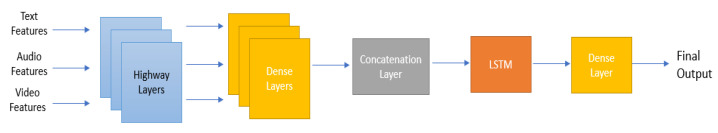
The LSTM model architecture.

The audio and video features were applied to those layers separately and then the outputs were concatenated with the text features. The final output of the model was obtained by feeding the concatenated features to the LSTM model. The model architecture consists of 3 feed-forward highway layers for gating, dense layers (sigmoid activation function) for dimensionality reductions, a concatenation layer, and an LSTM with 128 hidden nodes (the learning rate is 0.0001 and EarlyStopping call-back is used) (Figure 5).

F1-score, recall, precision, MSE, RMSE, MAE: We used the standard metrics for the evaluation of the model performance [89]. The precision gives the ratio of the actual positive cases to all predicted positive cases and tries to answer the question, ‘How many retrieved items are relevant?’ A high precision value is expected, and this indicates that the model has a low false positive rate. The recall gives the ratio of the actual positive cases to all cases with an answer ‘yes’ and tries to answer the question, ‘How many relevant items are retrieved?’ Again, it is expected that the recall value should be above 0.5. Another metric, the F1-score, shows the weighted average of the precision and recall metrics. Using this metric, all false positives and false negatives are considered. The F1-metric becomes a more useful measure than the accuracy when there is an uneven class distribution in the model.

(5) $$Precision=\frac{True\;Positives}{\left(True\;Positives+False\;Positives\right)}\;\;\;$$

(6) $$Recall=\frac{True\;Positives}{\left(True\;Positives+False\;Negatives\right)}\;\;$$

(7) $$F1\;Score=\;2*\frac{\left(Recall*Precision\right)}{\left(Recall+Precision\right)}$$

Other metrics, namely RMSE, MSE, and MAE, are defined in Equations (8)–(10). The MAE gives the mean value of the absolute values of each prediction error; the MSE mainly calculates the sum of the relative square error of each sample, while the RMSE is the square root of the MSE. While the RMSE and MSE show the standard deviation of the prediction errors, the MAE measures the average difference between predicted and actual depression values.

(8) $$RMSE=\sqrt{\frac{1}{n}\overset{n}{\underset{i=1}{\sum }}{\left(P_{i}-O_{i}\right)}^{2}\;\;\;}\;\;\;\;$$

(9) $$MSE=\frac{1}{n}\overset{n}{\underset{i=1}{\sum }}{\left(P_{i}-O_{i}\right)}^{2\;\;\;\;}\;$$

(10) $$MAE=\frac{1}{n}\overset{n}{\underset{i=1}{\sum }}\left|P_{i}-O_{i}\right|\;\;\;$$

For *n* number of interviews, *Pi* and *Oi* denote the predicted and actual depression values, respectively.

#### 3.2.4. Experimental Results

In this section, we carried out experiments on both the DAIC-WOZ and SymptomMedia datasets. We trained the models with the DAIC-WOZ dataset’s training+development sets and performed a depression classification for the DAIC-WOZ dataset’s testing set and the SymptomMedia dataset (Table 4). While evaluating the algorithms on DAIC-WOZ and SymptomMedia datasets, we extracted the same features from both. The features were those used in the original algorithms and are listed in Table 3. For the evaluation of all models (SVM+RF with SVM late fusion, SVM + RF with RF late fusion, LSTM without gating, and LSTM with gating), we provided the following tables and figures for the use of both bimodal and multimodal features with performance and error metrics.

Table 5 reports the performance of the SVM+RF with SVM late fusion model for the use of bimodal and multimodal features for both the DAIC-WOZ and SymptomMedia datasets. Although the use of multimodal features gave better results (F1-score: 0.46; MSE: 0.54) against the T + A and T + V bimodal features for the DAIC-WOZ dataset, it showed better performance (F1-score: 0.4; MSE: 0.57) against only the A + V bimodal features for the SymptomMedia dataset. The best recall value for SVM+RF with the SVM late fusion model was obtained by the A + V (0.55) and T + A (0.46) bimodal features for DAIC-WOZ and SymptomMedia datasets, respectively. However, the T+A+V multimodal features gave the highest precision value, 0.61, for the DAIC-WOZ dataset, and again the T + A bimodal features gave the highest precision value, 0.46, for the SymptomMedia dataset. In general, the A + V bimodal features showed a better performance for the DAIC-WOZ dataset and the T + A bimodal features showed a better performance for the SymptomMedia dataset.

Similar results for the comparison between the use of bimodal and multimodal features were found for the SVM + RF with RF late fusion (Table 6). The A + V bimodal features showed the best performance (F1-score: 0.56; MSE: 0.45) for the DAIC-WOZ dataset and the T + A bimodal features showed the best performance (F1-score: 0.46; MSE: 0.53) for the SymptomMedia dataset. The best recall and precision values for the SVM + RF with the RF late fusion model were obtained by the A + V (recall: 0.45; precision: 0.61) and T + A (recall: 0.46; precision: 0.46) bimodal features for the DAIC-WOZ and SymptomMedia datasets, respectively. In general, the A + V bimodal features showed better performance for the DAIC-WOZ dataset and the T + A bimodal features showed better performance for the SymptomMedia dataset, as did the SVM+RF with SVM late fusion model.

Table 7 reports the performance of the LSTM without gating model for the use of bimodal and multimodal features for both the DAIC-WOZ and SymptomMedia datasets. In general, this model gave better results than both the SVM + RF with SVM late fusion and SVM + RF with RF late fusion models. All modalities gave similar F1-scores, but the lowest MSE score (0.29) was given by the T+V bimodal features for the DAIC-WOZ dataset. For the SymptomMedia dataset, the use of multimodal features and the T + A bimodal features gave the same values for the F1-score (0.56) and all modalities gave similar MSE scores. For the DAIC-WOZ dataset, all modalities performed similarly according to the recall and precision values. However, for the SymptomMedia dataset, the T + A bimodal features and T + A + V multimodal features performed better, with the same F1-score (0.56), precision (0.58), and recall (0.57) values.

The best results for the use of multimodal features (F1-score: 0.64; MSE: 0.32) were obtained with LSTM with gating model for the DAIC-WOZ dataset (Table 8). Additionally, similar performance results (F1-score: 0.48; MSE: 0.34) were obtained with the use of multimodal features according to the use of bimodal features for the SymptomMedia dataset. Again, the T + A + V multimodal features performed well, with the best recall (0.66) and precision (0.94) values for the DAIC-WOZ dataset, while the T + A + V multimodal features performed the same with other modalities, with good recall (0.5) and precision (0.5) values for SymptomMedia dataset.

The highest F1-score (0.64) was obtained for the use of multimodal features with the LSTM with gating model for the DAIC-WOZ dataset, while the highest F1-score (0.56) was obtained for the use of multimodal features and T + A bimodal features with the LSTM without gating model for the SymptomMedia dataset. For the error metrics, the lowest MSE score (0.27) was obtained for the T + A bimodal features with the LSTM without gating model for the DAIC-WOZ dataset, and the lowest MSE score (0.34) was obtained for the use of multimodal features with the LSTM with gating model for the SymptomMedia dataset.

## 4. Discussion

### 4.1. Key Findings

The aim of this study was two-fold: first, to review the extent to which papers exploit different types of modalities in the implementation of depression classifications together with the types of AI models currently existing and the percentages of multimodal (text, audio, and video) data usage in the AI models in the literature (i.e., research question Q1); second, to explore the effects of multimodality and data bias with an experimental study as complementary work to the scoping review and to explain the need for explainable AI models (i.e., research questions Q2 and Q3).

Although there are some scoping reviews in the literature about the use of AI for mental health (including depression) [90,91] and depression recognition [92,93], to our knowledge, our scoping review is the first review investigating the different types of modalities used for depression classification with different AI models. Beyond our scoping review, we also conducted an experimental study and described its results. As such, our study has a unique position in the literature. The existing scoping reviews about depression recognition are mainly focused on using only one modality (facial [22], speech [25]) or using different observation techniques (rather than audio, text, and video) [94].

To address research question Q1, we identified 242 research papers or studies in total, of which only 20 (8.26%) analyzed all three groups of observable cues during depression classification. Most of the identified studies (179, 73.96%) in the literature still used a unimodal architecture that could detect only one aspect of depression. On the other hand, 38 (15.7%) studies used a bimodal architecture and 5 (2.06%) studies used a multimodal approach with physiological features.

Another important finding from the scoping review was related to the features that are used in multimodal classifications. The majority of the algorithms explore similar features, as AI algorithms for multimodal depression classification studies need to have a large volume dataset to obtain more accurate and less biased results. Powerful and accurate AI algorithms need more parameters and variables to feed their models, which means more data. Combining more data into your model helps in two ways: first, it provides more raw variables to be used as features, and second, it allows for new derived variables to be made by combining existing ones [95]. On the other hand, including all features in the models that we can find is not the aim of AI studies. The best possible feature set should be selected for the best result, but this discussion is beyond the scope of our review. All reviewed studies except two of them used the same dataset (DAIC-WOZ) for training and testing. This dataset has only 189 sessions. On the other hand, the variety of AI/ML techniques should be increased to increase the accuracy and decrease the error rate.

Another issue with multimodal depression classification studies is not having enough open-source codes for researchers, app developers, and product teams. Open source in an AI algorithm means that it is available for both commercial and non-commercial usage under certain licenses. Open source is the key element for innovation through collaboration. Open-source AI systems may include datasets, algorithms, and user interfaces. According to the latest technology report [96], nowadays 48% of enterprises are using open-source codes, and this number will increase to 65% within two years. Within our reviewed studies, only 3 (15%) studies provided their codes via GitHub. This may be one of the main reasons for the smaller number of multimodal depression classification studies in the literature.

A similar issue occurs regarding the types of reviewed studies. Only 7 (35%) studies were published in scientific journals, while the rest were published in conference proceedings. This result shows that there are still opportunities and open areas to contribute scientifically to multimodal depression classification.

To address research questions Q2 and Q3, we carried out experiments on the most widely used multimodal architectures, i.e., LSTMs, SVMs, and RFs. In the experimental study, we performed the depression classification process with four different models (SVM, RF, LSTM with gating, and LSTM without gating models) and four different modalities (T + A, T + V, A + V, and T + A + V).

The key finding of the experimental study was that there was an obvious benefit of analyzing multiple modalities during depression classification. The results clearly showed that multimodal approaches have the potential to outperform uni- and bimodal approaches. Overall, to answer research question Q2, the results showed that all multimodal architectures give superior or at least similar results to bimodal models (Table 6, Table 7 and Table 8).

It is easily observed that the negative effect of the small volume of datasets (data bias) is apparent and valid for all models. This is the main reason behind having lower performance rates and higher error rates. We provide a comparison table about the reported performance values of uni-, bi-, and multimodalities to emphasize the contribution of each modality to the depression classification process. According to the reported results, multimodal feature sets provide the best performance against the use of uni- or bimodal features in all studies (Table 9). To answer research question Q3, the results clearly show that the accuracy of the algorithms, especially those operating on uni- or bimodal assumptions, tends to degrade when introduced to recordings outside the nature of the training dataset. A more detailed analysis of the impact of using multiple modalities, especially in unfavorable datasets, is represented in the following section.

### 4.2. Challenges

#### 4.2.1. Decision Errors (Type I and Type II)

Misinterpretations or mistakes always exist within scientific studies because of their nature. The complete elimination of those decision errors is impossible, but controlling them is possible. There are two types of decision errors: false positives (type I errors), which show the decision as true while the actual case is false; and false negatives (type II errors), which show the decision as false when the actual case as true. To control and optimize these errors, we must understand the roles of false negatives and false positives specifically within our cases; while false negatives are preferable in some cases (e.g., criminal counts [97]), those errors can be very dangerous in different cases (e.g., clinical decisions [98], like in our case). If a patient with depression is not classified as depressed (false negative), then that patient may not get the proper clinical treatment and the mental disorder may get worse because of decision errors. False-positive cases for automatic depression classification have a less detrimental effect on people who have no depression at all and can be easily verified by other standardized tools such as questionnaires [99].

The F1-score is a more useful metric than other metrics because it considers the false negatives and is more meaningful when you have a non-symmetric dataset (Section 3.2.3.). On the other hand, the accuracy score gives the best performance when the burdens of the false negatives and false positives are similar. However, false negatives are more important than false positives concerning the consequences of different types of decisions in our case.

From the performance and error metrics tables (Table 3, Table 4, Table 5 and Table 6), the favorability of the datasets, variability between datasets, and variability among models can be compared according to the variability of the F1-scores. The A + T bimodal features were the only features that showed improvements for all four models between datasets. The A+V bimodal features showed the most variation (more data bias) in a negative direction, with the results varying depending on the favorability of the dataset. Our testing dataset, the SymptomMedia dataset, which is a high-quality dataset with high expressivity of the features, might be favorable for the bimodal features for SVM and RF approaches (higher F1-scores), and this shows that if the features are not so distinct within the dataset (the SymptomMedia dataset has higher F1-scores than the DAIC-WOZ dataset), the F1-score will significantly decrease, which is not reliable at all. On the other hand, the multimodal features showed minimal F1-score variations for all four models, which means that the usage of multimodal features gives more resilient and reliable results. When we compare the models, the SVM and RF models generated less variability than the other models.

#### 4.2.2. Data Bias

The data bias is one of the important challenges for machine learning models. These models lack visibility because of having a ‘black box’ structure. If we are trying to solve a problem in the healthcare sector and want to use AI, the paramount factor to consider is the data itself. The data are the main and only source of bias, which tends to be influenced by multiple factors such as the patient population and the correlation between parameters [58]. Training without considering those factors results in the misrepresentation and misunderstanding of the phenomenon. The other source of data bias is not having enough data in the health domain, as in other fields such as computer vision and data mining. Even in our scoping review, the sizes of the datasets were not sufficient (DAIC-WOZ, 189 subjects; BDC WB, 51 subjects; AViD-C, 84 subjects). We selected those studies because they contained only multimodal depression features. Besides those features, other features can be gathered and added to the model from different devices such as EEG, ECG, and fMRI devices. If we take into account all of those features, then those may lead to another problem called ‘the curse of dimensionality’ [100]. In Section 3.1.1 and Section 3.1.2 methods, the LDA, PCA, clustering, and RELIEFF approaches are highlighted as practical solutions to at least partially overcome the challenge of dimensionality and to generate optimal sets of features.

Beyond those issues, another problem that causes bias with the DAIC-WOZ dataset is having binary labels based on PHQ-8 scores from the participants’ self-reports. These self-reports may depend on social, subjective, and other kinds of biases, and may affect the annotation accuracy [101]. Thus, taking the DAIC-WOZ dataset as a ground truth may not be the most accurate way to measure the depression status. As a result, those issues can be counted as additional potential reasons for the low performance values.

One of the ways to overcome these challenges is to use explainable and interpretable models. Explainable AI models provide transparency, fairness, and reliability in the process and results. The mitigation of bias in the model includes removing the effect of the bias. There are different techniques for mitigation that can be applied in each phase of the model (pre-processing, in-processing, and post-processing). For example, while the reweighing technique [102] involves changing the weights of the features before the training in the pre-processing phase, the adversarial debiasing technique [103] involves applying an optimization process using a GAN during the training process. On the other hand, the techniques used after the training phase have less pronounced effects. For example, the equalized odds [104] technique involves adjusting the thresholds to obtain smaller differences between the true and false results. In summary, it is shown that explainable AI techniques can be used for bias detection and that there are some useful tools that can be used for this, such as a fairness report [105], SHAP [106], and CERTIFAI [107].

### 4.3. Limitations of the Scoping Review

While this scoping review provides comprehensive insight into the cues of depression, which are observable from text, audio, and video sources, and contributes to the literature by presenting an additional experimental study to show the importance and effects of the use of multimodal features for depression classification, it does not provide a complete picture of the current state of the research on depression classification due to certain limitations. First, our scoping review did not include other observable features of depression from different devices (i.e., ECG, EEG, fMRI). Although those features may also contribute to the classification of depression, we may again be faced some problems related to the existing dataset and feature dimensionality. Second, we focused only on papers written in English. The studies in other languages may include additional information about the classification of depression. Third, we tried to show which method tends to be more resilient to data collected outside the training datasets in the experimental part of our study. Although the SymptomMedia recordings are not exactly categorized as real-world data, the data are of a different nature, and from the psychological perspective represent a high-quality simulation of real-world data. Fourth, as specified in the Discussion, one of the main issues of ML in the health domain is the volume of available datasets. In our experiments, we trained our models with 142 cases and tested them with 75 cases. Thus, our results, just like those of the reviewed studies, need to be validated with larger datasets. In the future, if new depression datasets emerge and are available as open-source data, we will try to increase the sizes of both the training and testing sets.

## 5. Future Work

This scoping review illustrates the state of the existing studies, their limitations, and the future directions for multimodal depression classification. More studies on multimodal depression classification should be carried out in the future to show the different aspects of the observable cues of depression. To increase the accuracy rates of the classification algorithms, high-volume depression datasets should be constructed and made available for researchers. With the advances in omics, genetics, and epigenetics, these datasets have become valuable and affordable sources of real-world data in multiple studies of depression classification [29,108]. With a stable and more distinct representation of depressive disorders, these data could significantly increase the discriminatory power of the AI algorithms. Although less accessible at the moment, future studies should analyze the correlation between genetic or epigenetic and observable features of depression. Furthermore, there is a lack of explainable artificial intelligence algorithms, which would significantly improve the interpretability of the results and expedite their integration into clinical routine. Additionally, explainable AI or at least hybrid model algorithms may offer a way to mitigate the data quantity requirements of deep models and to exploit the natural relationships (or interplay) between the modalities, as observed by experts in real-world manifestations of depression. The future research should focus more on the multimodal nature of human interactions and the explainability of AI decisions, as multimodal techniques are, according to our findings, more resilient to data bias. Finally, further clinical studies should be carried out to evaluate the performance of the algorithms in real-world settings, with real patients, and to compare the algorithms against standardized instruments.

## Figures and Tables

**Table 1:** The final set of 20 papers that passed the eligibility criteria and were reviewed in detail.

No	Reference	AI/ML Technique	Reported Performance Value	Dataset	Feature Fusion	Github Code	Type
1	Yang et al., 2021 [41]	CNN, SVM, RF	F1: 0.74	DAIC-WOZ	Early/Late	No	Journal
2	Samareh et al., 2018 [42]	RF	RMSE: 4.35	DAIC-WOZ	Late	No	Journal
3	Prabhu t al., 2021 [43]	CNN, LSTM	F1: 0.75	DAIC-WOZ	Late	No	Journal
4	Ceccarelli and Mahmoud, 2021 [44]	LSTM	F1: 0.87	BDC-WB	Late	Yes	Journal
5	Shan et al., 2020 [28]	SVR and RF	RMSE: 4.96	DAIC-WOZ	Early	No	Journal
6	Dham et al., 2017 [45]	SVM and NN	RMSE: 5.35	DAIC-WOZ	Late	No	Journal
7	Qureshi et al., 2019 [46]	CNN	F1: 0.48	DAIC-WOZ	Early	No	Journal
8	Ray et al., 2019 [47]	LSTM	RMSE: 4.28	DAIC-WOZ	Late	No	Proceeding
9	Williamson et al., 2016 [48]	MV-R	F1: 0.81	DAIC-WOZ	Late	No	Proceeding
10	Yang et al., 2016 [49]	DT	F1: 0.72	DAIC-WOZ	Early	No	Proceeding
11	Yang et al., 2017 [50]	CNN	RMSE: 4.65	DAIC-WOZ	Late	No	Proceeding
12	Yin et al., 2019 [51]	LSTM	RMSE: 5.50	DAIC-WOZ	Early	No	Proceeding
13	Rohanian et al., 2019 [52]	LSTM	F1: 0.81	DAIC-WOZ	Early/Late	Yes	Proceeding
14	Gong, 2018 [53]	SVM and RF	F1: 0.6	DAIC-WOZ	Early	No	Proceeding
15	Gupta et al., 2014 [54]	LR	Corr. Coef: 0.52	AViD-C	Early/Late	No	Proceeding
16	Pampouchidou et al., 2016 [55]	DT	F1: 0.66	DAIC-WOZ	Early/Late	No	Proceeding
17	Sun et al., 2017 [56]	RF	RMSE: 4.7	DAIC-WOZ	Late	No	Proceeding
18	Morales et al., 2018 [57]	SVM	F1: 0.49	DAIC-WOZ	Early/Late	No	Proceeding
19	Haque et al., 2018 [58]	CNN	Sens: 83.3%; Spec: 82.6%	DAIC-WOZ	Early	Yes	Proceeding
20	Qureshi et al., 2021 [59]	LSTM	RMSE: 3.49	DAIC-WOZ	Late	No	Proceeding

**Table 2:** The selected SymptomMedia films.

No.	Recording Title	Depression Status
1	Depression Assessment A-1	D
2	Depression Assessment A-2	D
3	Depression Assessment A-3	D
4	Major Depressive Disorder A-1	D
5	Major Depressive Disorder Recurrent Episode Severe Part 1	D
6	Major Depressive Disorder Recurrent Episode Severe Part 2	D
7	Major Depressive Disorder Recurrent Episode Severe Part 3	D
8	Major Depressive Disorder Recurrent Episode Severe Part 4	D
9	Major Depressive Disorder with Anxious Distress	D
10	Major Depressive Disorder with Melancholic Features	D
11	Major Depressive Disorder with Peripartum Onset	D
12	Patients with Depression A-1	D
13	Major Depressive Disorder with Seasonal Pattern	D
14	Major Depressive Disorder	D
15	Narcissistic Personality Disorder	ND
16	Antisocial Personality Disorder Version 1	ND
17	Histrionic Personality Disorder Version 2	ND
18	Stimulant Use Disorder	ND
19	Schizoid Personality Disorder	ND
20	Schizotypal Personality Disorder	ND
21	Alcohol Use Disorder	ND
22	Anorexia Nervosa Binge-Eating Purging	ND
23	Antisocial Personality Disorder Version 2	ND
24	Avoidant Personality Disorder	ND
25	Binge Eating Disorder	ND
26	Bulimia Nervosa	ND
27	Dependent Personality Disorder	ND
28	Histrionic Personality Disorder Version 1	ND

Notes: D = diagnosis of depression; ND = no diagnosis of depression.

**Table 3:** The details of the extracted features.

Modality	Category	Features	Descriptions
Audio	Prosodic	F0	Fundamental frequency created by the vibrations in the vocal cords
		VUV	Binary decision of voicing—unvoicing part
		Energy	Amplitude variation of speech signals over time (volume or intensity)
		Duration	Amount of time to build vowels, words, and similar constructs
	Spectral	MFCC	Mel Frequency Cepstral Coefficients: Short-term power spectrum of the speech signal.
		HMPDM0-24	Harmonic Model and Phase Distortion mean
		HMPDD0-12	Harmonic Model and Phase Distortion deviations
		LPCC	Linear Prediction Cepstral Coefficients
		LFPC	Log-Frequency Power Coefficients
		GFCC	Gammatone Frequency Cepstral Coefficients
		Formants	Frequencies of the acoustic resonance of the vocal tract
	Voice quality	NAQ	Normalized amplitude quotient
		QOQ	Quasi-open quotient
		H1H2	Differentiated glottal source spectrum
		PSP	Parabolic spectral parameter
		MDQ	Maxima dispersion quotient
		peakSlope	Spectral tilt/slope of wavelet responses
		Rd	Shape parameter of the Liljencrants–Fant model of the glottal pulse dynamics
		jitter	variability of fundamental frequency between successive vibratory cycles
		shimmer	variable of the amplitude between successive vibratory cycles
		Harmonics to Noise Ratio	relative level of noise in the frequency spectrum of vowels
		Teager Energy Operator	used to detect stress in speech
Video/Image	Action Units	AU01- 02/04- 06/09- 10/12/14- 15/17/20/25- 26_r	Regression values of Inner brow raiser, Outer brow raiser, Brow lower, Upper lid raiser, Cheek raiser, Nose wrinkle, Upper lip raiser, Lip corner puller, Dimpler, Lip corner depressor, Chin raiser, Lip stretched, Lip part, Jaw drop
		AU04/12/15/23/28/45_c	Binary outputs of Brow lower, Lip corner puller, Lip corner depressor, Lip tightener, Lip suck, Blink
	Eye gaze	x_0, y_0, z_0, x_1, y_1, z_1	Vectors of both eyes in world coordinate space
		x_h0, y_h0, z_h0, x_h1, y_h1, z_h1	Vectors of both eyes in head coordinate space
	Head pose	pose_confidence/success	pose_confidence/success
		Tx, Ty, Tz	Vectors of head in world position coordinate space
		Rx, Ry, Rz	Vectors of head in head rotation coordinate space
	Facial Landmarks	68 2D points on face.	The points are in pixel coordinates.
		68 3D points on face.	The points are in millimetres in the world coordinate space
Text	Lexical	Word2Vec	300-feature vector representation (sentences × words × 300 features)

**Table 4:** The sizes of the datasets.

	DAIC-WOZ	SymptomMedia
Testing Set	47	28
Development Set	35	-
Training Set	107	-

**Table 5:** The performance and error metrics for the SVM + RF with SVM late fusion (T + A: text and audio; T + V: text and video; A + V: audio and video; T + A + V: text, audio, and video).

	DAIC-WOZ DB Dataset						SymptomMedia Dataset					
**Modality**	**F1-Score**	**Recall**	**Precision**	**MSE**	**RMSE**	**MAE**	**F1-Score**	**Recall**	**Precision**	**MSE**	**RMSE**	**MAE**
**T + A**	0.38	0.39	0.55	0.61	0.78	0.61	0.46	0.46	0.46	0.53	0.73	0.53
**T + V**	0.42	0.41	0.55	0.59	0.76	0.59	0.42	0.43	0.42	0.57	0.75	0.57
**A + V**	0.56	0.55	0.6	0.45	0.67	0.45	0.37	0.39	0.38	0.6	0.77	0.6
**T + A + V**	0.46	0.45	0.61	0.54	0.73	0.54	0.4	0.43	0.41	0.57	0.75	0.57

**Table 6:** The performance and error metrics for the SVM + RF with RF late fusion (T + A: text and audio; T + V: text and video; A + V: audio and video; T + A + V: text, audio, and video).

	DAIC-WOZ DB Dataset						SymptomMedia Dataset					
**Modality**	**F1-Score**	**Recall**	**Precision**	**MSE**	**RMSE**	**MAE**	**F1-Score**	**Recall**	**Precision**	**MSE**	**RMSE**	**MAE**
**T + A**	0.38	0.39	0.55	0.61	0.78	0.61	0.46	0.46	0.46	0.53	0.73	0.53
**T + V**	0.42	0.41	0.55	0.59	0.76	0.59	0.42	0.43	0.42	0.57	0.75	0.57
**A + V**	0.46	0.45	0.61	0.54	0.73	0.54	0.3	0.36	0.29	0.64	0.8	0.64
**T + A + V**	0.44	0.43	0.59	0.56	0.75	0.56	0.4	0.43	0.41	0.57	0.75	0.57

**Table 7:** Performance and error metrics for the LSTM without gating (T + A: text and audio; T + V: text and video; T + A + V: text, audio, and video).

	DAIC-WOZ DB Dataset						SymptomMedia Dataset					
**Modality**	**F1-Score**	**Recall**	**Precision**	**MSE**	**RMSE**	**MAE**	**F1-Score**	**Recall**	**Precision**	**MSE**	**RMSE**	**MAE**
**T + A**	0.53	0.57	0.51	0.27	0.52	0.48	0.56	0.57	0.58	0.37	0.61	0.47
**T + V**	0.52	0.55	0.5	0.29	0.54	0.49	0.45	0.46	0.46	0.36	0.6	0.48
**T + A + V**	0.53	0.55	0.52	0.35	0.59	0.51	0.56	0.57	0.58	0.37	0.6	0.47

**Table 8:** The performance and error metrics for the LSTM with gating (T + A: text and audio; T + V: text and video; T + A + V: text, audio, and video).

	DAIC-WOZ DB Dataset						SymptomMedia Dataset					
**Modality**	**F1-Score**	**Recall**	**Precision**	**MSE**	**RMSE**	**MAE**	**F1-Score**	**Recall**	**Precision**	**MSE**	**RMSE**	**MAE**
**T + A**	0.45	0.43	0.57	0.33	0.58	0.48	0.48	0.5	0.5	0.35	0.59	0.44
**T + V**	0.42	0.41	0.55	0.35	0.59	0.49	0.5	0.5	0.5	0.38	0.61	0.47
**T + A + V**	0.64	0.66	0.64	0.32	0.57	0.45	0.48	0.5	0.5	0.34	0.58	0.44

**Table 9:** Comparison table for the reported performance values for all modalities (T: text; A: audio; V: video).

Study	Reported Performance Values for Uni-/Bi-/Multimodalities			
Shan et al., 2020 [28]	**T**	**T + A**	**T + V**	**T + A + V**
	RMSE: 5.58, MAE: 4.41	RMSE: 5.3(SVR), MAE: 3.94	RMSE: 5.44, MAE: 4.24	RMSE: 4.96, MAE: 3.84
Dham et al., 2017 [45]		**T + A**	**V**	**T + A + V**
		RMSE: 5.98, MAE: 4.37	RMSE: 5.37, MAE: 4.65	RMSE: 5.35, MAE: 4.37
Qureshi et al., 2019 [46]	**T**	**A**	**V**	**T + A + V**
	RMSE: 4.7, MAE: 3.81	RMSE: 6.55, MAE: 5.67	RMSE: 6.28, MAE: 5.03	RMSE: 4.24, MAE: 3.29
Ray et al., 2019 [47]	**A + V**	**T + A**	**T + V**	**T + A + V**
	RMSE: 5.38	RMSE: 4.37	RMSE: 4.64	RMSE: 4.28
Williamson et al., 2016 [48]	**T**	**A**	**V**	**T + A + V**
	RMSE: 4.46, MAE: 3.34	RMSE: 6.38, MAE: 5.32	RMSE: 6.45, MAE: 5.33	RMSE: 5.31, MAE: 4.18
Samareh et al., 2018 [42]	**T**	**A**	**V**	**T + A + V**
	RMSE: 5.86, MAE: 4.88	RMSE: 5.89, MAE: 5.18	RMSE: 5.65, MAE: 4.87	RMSE: 4.78, MAE: 4.05
Pampouchidou et al., 2016 [55]	**T**	**A**	**V**	**T + A + V**
		F1: 0.59 (0.87)	F1: 0.5 (0.9)	F1: 0.62 (0.91)
Haque et al., 2018 [58]	**T**	**A**	**V**	**T + A + V**
	TPR: 57.5, TNR: 60.3	TPR: 53.9, TNR: 54.6	TPR: 61, TNR: 59.7	TPR: 74.2, TNR: 76.1
Qureshi et al., 2021 [59]	**T**	**A**	**V**	**T + A + V**
	MSE: 24.02, MAE: 4.09	MSE: 41.96, MAE: 5.19	MSE: 44.29, MAE: 5.23	MSE:22.25, MAE: 3.49

## Data Availability

The dataset used and analyzed during the current study is available from the corresponding author on reasonable request. The DAIC-WOZ database can be obtained from the original authors (https://dcapswoz.ict.usc.edu/daic-woz-database-download/ accessed on 6 April 2022). The SymptomMedia dataset can be obtained from the original authors (https://symptommedia.com/ accessed on 31 March 2022). Please refer to the websites for more information.

## List of Abbreviations

AI	Artificial Intelligence
AUs	Action Units
AVEC2016	Audio Visual Emotion Challenge
AViD-C	AViD-Corpus
BDC-WB	Bipolar Disorder Corpus and Well-Being
BiLSTM	Bidirectional Long Short-Term Memory
CNN	Convolutional Neural Network
DAIC-WOZ	Distress Analysis Interview Corpus Wizard-of-Oz
DSM 5	The Diagnostic and Statistical Manual of Mental Disorders, Fifth Edition
DT	Decision Tree
ECG	Electrocardiogram
EEG	Electroencephalography
fMRI	Functional Magnetic Resonance Imaging
ICD 10	The International Classification of Diseases Tenth Edition
LSTM	Long Short-Term Memory
MAE	Mean Absolute Error
ML	Machine Learning
MSE	Mean Square Error
MV-R	Multivariate Regression
NN	Neural Network
PHQ-8	The Eight-Item Patient Health Questionnaire Depression Scale
PRISMA-ScR	(Preferred Reporting Items for Systematic Reviews and Meta-Analyses—Extension for Scoping Reviews)
QoL	Quality of Life
RF	Random Forest
RMSE	Root Mean Square Error
SVM	Support Vector Machines
TPR/TNR	True Positive Ratio/True Negative Ratio
WOS	Web of Science
